# ‘This is the beginning of the new me’: process evaluation of a group fitness intervention to promote wellbeing in formerly homeless individuals

**DOI:** 10.1186/s12889-018-5175-5

**Published:** 2018-02-27

**Authors:** Ernesta Sofija, Melanie Plugge, Nicola Wiseman, Neil Harris

**Affiliations:** 1 School of Medicine, Griffith University, Gold Coast Campus, Gold Coast, Australia; 2 Menzies Health Institute Queensland, Griffith University, Gold Coast Campus, Gold Coast, Australia

**Keywords:** Group fitness intervention, Homeless wellbeing, Social integration, Supportive housing, Lifestyle

## Abstract

**Background:**

Homelessness is a persistent social issue with diverse impacts reaching far beyond individuals. Strategies and research concerning homelessness and health have largely focused on the risk factors and weaknesses of individuals. Such preoccupation has meant the potential strengths and resources within individuals, and so-called strength-based approaches have received less attention. Consequently, understanding how to effectively work with and engage this population in such interventions is limited.

**Methods:**

The current study presents a process evaluation of an 8-week group fitness intervention in a supportive housing facility. The purpose of the intervention was to increase tenants’ physical activity together with opportunities for social interaction and support to, in turn, improve physical and mental wellbeing, and ultimately help individuals re-engage in their community. The evaluation focused on seven key components: context, recruitment, reach/participation, dose delivered, dose received, satisfaction/feedback and fidelity. Data collection methods included observation, attendance records and participant and staff interviews.

**Results:**

Findings indicate the intervention was appropriate, well delivered, and enjoyed by participants who highlighted the importance of the sessions for their mental wellbeing and social inclusion. The intervention being conducted on site, the trainers’ ability to build good rapport with participants together with the supportive environment they created were central to successful implementation.

**Conclusion:**

Group fitness sessions represent a promising intervention to improve wellbeing of this population. However, the need for more personalised care when delivering fitness sessions, due to the complexity of health issues prevalent in this population, was identified. This has implications for already limited resources, including staffing. Strategies to address this are required to ensure the continuity of fitness programs. Impact evaluation to quantify changes/improvements in wellbeing would complement this work and add much to understanding the effects of participation.

## Background

Homelessness is a significant population health issue in Australia and internationally with the number of homeless individuals steadily increasing since the 1980s [1, 2]. On any given night in Australia, approximately 105,000, or 1 in 200, individuals experience homelessness [3, 4]. According to the Australian Bureau of Statistics, an individual is considered homeless if she/he does not have suitable accommodation alternatives, when the current living arrangement is: ‘in a dwelling that is inadequate; or has no tenure, or if their initial tenure is short and not extendable; or does not allow to have control of, and access to space for social relations’ [5]. Homelessness, however, is not simply an issue of a lack of safe shelter, it is a condition of detachment from society with far-reaching implications on individual health and wellbeing [6]. The homeless population is also accountable for a disproportionate use of social, judicial and healthcare resources, and ultimately poses a significant economic cost to society [1]. For example, in Australia, total expenditure for youth homelessness only is about $626 million per year [7].

Homeless people have a significantly higher mortality rate, with an average life expectancy ranging from 42 to 52 years, compared to approximately 80 years for the general population in Western countries like United States of America and United Kingdom [8, 9]. Reduced life expectancy of the homeless can largely be explained by high prevalence of morbidity, which can precede or be a consequence of being homeless. A study conducted in Brisbane showed that 50% of homeless people display a tri-morbidity of physical illness, mental health issues and substance abuse [10]. This population frequently suffers from the negative consequences of alcohol and drug abuse, such as violence, sexual abuse and infectious disease [1]. Homelessness itself is an independent risk factor for premature death [11]. For example, compared to the most deprived populations based on socio-economic indicators, homeless people are at greater risk of dying prematurely from specific causes such as drug related conditions, respiratory disorders and circulatory diseases [11].

Even though homeless people represent some of the most vulnerable and socially excluded members of society, they commonly find it especially challenging to access the help they require, and often struggle to find or do not seek opportunities to connect with others [12, 13]. This is due to a number of complex and interrelated reasons, such as stigma [14], social exclusion [15], coupled with considerable burden of cognitive dysfunction [16]. This results in further disadvantage to an already vulnerable population due to social exclusion, which is recognised as one of the key reasons for the subjective wellbeing of homeless people being lower than that of general population [15].

Interventions that foster experiences enabling homeless people to form multiple group memberships provide opportunities for homeless people to build strengths such as social capital, leading to long-term wellbeing [15]. This demonstrates that strategies promoting the development of social networks and support can alleviate negative effects of discrimination resulting from homelessness, improve wellbeing, and facilitate reintegration into society [17–19]. Interestingly, to date, such strategies have been largely overlooked often in favour of strategies targeting employment, addictive substance use and housing. Little research has been published regarding how to effectively implement such interventions with this population [18]. Considering the complexity of issues faced by homeless or formerly homeless individuals, addressing this gap is a crucial step in developing and implementing effective interventions. A process evaluation can help to identify unexpected challenges and complications, which compromise the effectiveness of much needed interventions [20].

This paper reports the findings of a process evaluation that examined the implementation of a pilot group fitness intervention implemented in a supportive housing facility in Queensland. Supportive accommodation provides a platform to engage with this population to improve their wellbeing [15]. The intervention aimed to increase physical activity, improve physical and mental health and encourage social interaction and support among participants, as a way to build strengths within individuals themselves, and, in turn, help them re-engage with their community. In addition to well established physical health benefits, exercise has been shown to improve cognitive ability, mental health, and general quality of life [21–23]. Exercise has also been linked to increased self-efficacy and confidence, which results in improved self-care and ultimately a higher likelihood of individuals connecting with friends and family members, hence improving social health [24, 25]. Participation in group fitness activities enable participants to meet others that share common interests, which can result in the formation and strengthening of friendships and support networks [26]. Additionally, it could help reduce marginalisation, and help tenants re-engage in other areas of their lives, such as education and employment [26, 27].

While academic literature on physical activity among homeless people is sparse, available research indicates that physical activity interventions can be appropriate and particularly beneficial for this vulnerable population [28, 29]. Research shows that the prevalence of modifiable health risk factors such as alcohol abuse, smoking and physical inactivity in this population is high [11, 29, 30]. Studies have documented physical activity rates less than recommended levels ranging from 30% to 45.3% together with poor physical flexibility [28–31]. Further, research suggests that homeless people desire the physical, emotional and spiritual benefits associated with physical activity, with some perceiving it as a method to self-management of mental health [28, 29]. Nevertheless, only a few physical activity interventions have been implemented for this population with some promising results such as improvements in social capital, fitness and social networks, increased physical activity, and reduced substance use [31–33]. The reported research also acknowledges challenges of working with this complex population highlighting the need to build a better understanding of how to work with this population to inform the development of population sensitive and appropriate interventions [33].

The aim of this process evaluation was to better understand how to effectively work with this population in lifestyle related strength-based interventions. The findings contribute to existing literature by providing guidance as to how to implement such programs and to develop recommendations to inform future development of this and similar interventions.

## Methods

### Setting

The group fitness intervention took place in a supportive housing facility located in South-east Queensland, Australia with approximately 150 tenants, of whom at least half have experienced chronic homelessness with the rest being at risk of homelessness or having very low to low income. The housing facility offers long term affordable housing, and includes various support services, such as physical and mental health practitioners, support workers, security guards, nutritionists and various types of volunteers. Tenants have complex physical, social and mental health concerns, and some suffer from the effects of drug and alcohol abuse.

### Study participants

Participants included tenants who attended group fitness sessions, as well as the exercise physiologists and the project overseers. Participant physical capacity to engage in group fitness sessions was determined by the qualified exercise physiologists delivering the intervention. Of the 24 tenants who participated in the intervention, 18 consented to be part of the process evaluation. Convenience sampling was used to recruit the 18 tenants (11 females; 7 males).

Four project staff members were interviewed about their involvement in the project and their experience during the intervention, including two exercise physiologists (worked directly with clients to deliver the fitness sessions), one supportive housing staff member (who was involved in the initiation of the project and regularly checked in on participants and project progress), and one project overseer (who was involved with the start-up of the project including sourcing the trainers and equipment for the facility).

### The intervention

The pilot intervention incorporated 8 weeks of regular group fitness sessions delivered by qualified exercise physiologists. The group fitness sessions were delivered in a fitness and wellbeing centre (hereafter fitness room), which is a 20-square metre detached on-site building that had been converted from an unused space into a fitness room. The intervention, established in consultation with tenants, sought to improve mental and physical health of tenants, improve social connectedness and overall wellbeing. The ultimate goal of the intervention was to instigate change by building strengths/resources, and help tenants re-engage in their community and other areas of their lives, such as education and employment.

The fitness room was open Monday to Friday at 10:00 and 15:00 for one-hour group fitness sessions. Participation in the fitness sessions was free, and tenants were able to attend as many sessions per week as they liked. Exercise physiologists were informed of participants’ mental and physical health conditions, and tailored exercise routines specifically to individual capabilities. At least one trainer was on site per session to assist the tenants.

### Data collection methods

The evaluation was guided by the theoretical framework for process evaluation adapted by Steckler and Linnan [34], which included seven key components of evaluation: context, recruitment, reach/participation, dose delivered, dose received, satisfaction/feedback and fidelity of the project. Evaluation questions that align with each component, and the levels of sources (staff/organisation and participant) for the data collected are presented in Table 1. A mixed methods approach was used to collect data, methods used included:

**Table 1:** Process evaluation components and questions, levels and data collection methods

Component	Evaluation questions	Data collection methods	Staff level/ Organisation	Participant level
Context	• What factors in the organization, community, social/political environment, or other situational issues could potentially affect intervention implementation?	• Observations using checklist • Semi-structured interviews	x	x
Recruitment	• What strategies were used to recruit participants? • What were the barriers to maintaining involvement of individuals?	• Observations • Semi-structured interviews	x	
Reach/Participation	• What proportion of the target population participated in the intervention and in each program session? • Characteristics of intervention participants • What were the perceived reasons for non-participation?	• Daily participant attendance records • Observations using checklist • Semi-structured interviews	x	x
Dose delivered	• To what extent were the intended activities provided to the participants? • To what extent were the resources, including equipment and staffing used? • To what extent were all of the intended strategies and activities used?	• Observations, • Semi-structured interviews	x	
Dose received	• To what extent participants, present in the fitness sessions, were engaged in the activities? • How did participants react to trainers, equipment and other participants?	• Observations • Semi-structured interviews	x	x
Satisfaction/feedback	• Did participants enjoy participating in group fitness activities? • How did they feel after the sessions? • What motivated them to participate?	• Observations • Semi-structured interviews	x	x
Fidelity	• To what extent was the intervention implemented consistent with the underlying theory and philosophy? • Did exercising in the group setting promote social interaction?	• Observations • Semi-structured interviews	x	x

**Daily participant attendance records:** kept by the exercise physiologists on site.

#### Observation reports

Fitness sessions were observed using an observation checklist**.** Documentation of physical surroundings, activities conducted, resources used, implementation, participation levels, social interactions, trainer capability and general participant feedback were recorded. Additionally, date and time of observation, number of participants attending, and a general summary of each session were recorded.

#### Semi-structured interviews with participants

Post exercise session interviews were conducted to explore enjoyment of activities, perceived benefits, feedback on intervention implementation and resources, participation and motivation to participate. These weekly formative interviews were conducted as participants exited the fitness room at the conclusion of sessions. The interviews, often conducted with small groups of participants, were purposefully short to capture their views in the moment while not impinging upon their enjoyment of post exercise euphoria or the flow of their day. This approach, while appropriate for the population of interest, made it difficult to assign “sound bites” to individuals, and therefore quotes were recorded by week of intervention.

#### Semi-structured interviews with staff

In-depth interviews with four staff members and stakeholders were conducted to understand the context, project induction process, challenges faced, implementation including use of resources, perceived effectiveness of intervention, recruitment process, participation levels and barriers to attendance, any noticeable participant changes since implementation, and suggestions for improvement.

The selection of methods was made in consultation with the industry partners and with due consideration of the characteristics of the population of interest including the desire to minimise burden upon participants. The methods were considered sensitive to the population and matched to the purpose of the research to examine process of implementation.

### Data analysis

Descriptive analyses were conducted to summarise participant attendance records. Interviews were recorded and transcribed into a Microsoft Word Document. A two-stage analysis process was adopted. First, transcripts were coded by three independent researchers (ES, NH and MP) according to the components of the process evaluation framework of Steckler and Linnan [34]: context, recruitment, reach/participation, dose delivered, dose received, satisfaction/feedback, and fidelity. This framework had been used to guide data collection. Second, the data under each component was analysed and in several instances data was organised under sub-components.

## Results

### Context

This study aimed to analyse the context, which includes the factors in the organization, community, social/political environment, or other situational issues that could potentially affect intervention implementation. Findings around physical space and broader context in which the intervention was implemented are presented below.

### Physical space

The facility contained 20-square metres of exercise space, which remained tidy and organised for the duration of the intervention. Generally, the facility size was suitable for the intervention with the exception of instances when the number of participants exceeded 5 people, causing some to exercise outside. This also reflects in participant comments made during the interviews:

*‘I prefer a smaller group of participants because the room can get crowded. It would be nice to have more space* (Week 5).*’*

The space was improved throughout the intervention. For example, when hot temperatures resulted in discomfort a fan was installed, after which exercising became more comfortable for participants. A music device was introduced to give participants a chance to play their favourite music, thus encouraging motivation and participation. The facility was located next to a construction site, and the noise levels occasionally distracted participants and led to complaints. However, many participants commented that they appreciated the positive and cheerful environment, and convenience of the facility, all of which motivated participants to attend:

*‘I’m really enjoying the exercises. It’s a good environment, the construction noise is just a little distracting* (Week 2).*’*

*‘This facility is a good standard* (Week 2)*.’*

*‘Pain. I don’t want it. I want to be fit. Exercise on its own isn’t very exciting. This is so close and convenient - why wouldn’t I come* (Week 5)*.’*

*‘I can trust that if I’m feeling down if I just get myself to the door (fitness facility) it’ll all be fine. I trust there won’t be any judgement there and there never is* (Week 5)*.’*

*‘It’s in the building, it’s convenient, it’s a friendly environment, and I like knowing that I’m doing something positive* (Week 8).*’*

According to project staff, the space matched with equipment was suitable for the intervention, and was respected by the participants.

*‘Thera-bands and dumbbells, Swiss balls, yoga mats, the magic circle/Pilates ring. They’re our go to pieces of equipment because they’re very versatile. In the space that we’ve got they allow for a lot of different programming to happen* (Staff 3).*’*

*‘I think it’s a very well respected place. They [participants] can understand the value of it* (Staff 3).*’*

### Broader context

The intervention was implemented in Brisbane Common Ground, a supportive housing facility owned by the Department of Housing and Public Works, in collaboration between three organisations; Common Ground Queensland (not-for-profit organisation) and Rise Industries (Proprietary Limited) were involved in the initiation and development of the project, including the establishment of the fitness room, and Iridium Health (Proprietary Limited) that joined the project later to deliver the fitness sessions. A detached building on the facility premises was not being used. A tenant-based planning process initiated to facilitate better usage of some of the building’s ground floor common areas instigated the development of the fitness room with majority of participants indicating they preferred it to be converted into such a facility. However, securing funding to establish the fitness room proved difficult.

*‘(…), and it was a long process tracking it and getting a donation to renovate it. We did submit a grant 3 times to Brisbane City Council. We had some great feedback. They asked us to tweak things and resubmit but after the third time of not securing that we decided to go down a different track, and that’s when an ex-board member decided to give us a donation. So that came out of the blue. Then we applied for a Suez Environment Community Grant which paid for most of the equipment that’s in there* (Staff 1)*.’*

There was no funding available for intervention staffing. Sourcing trainers was identified to be one of the key challenges that resulted in delays to the project commencement. Due to the complexity of health, social and other wellbeing issues faced by the target population, trainers needed to have niche skill set as well as experience working with vulnerable populations. Having no budget for human resources required finding trainers who would commit to working on a voluntary basis, which was another factor that substantially reduced the pool of potential candidates.

*‘The room was ready to go and we had a fitness instructor sourced at the opening, but we had a few issues around the suitability of that person, and that person decided to pull out* (Staff 1)*.’*

*‘It was a struggle to actually get someone engaged, not just with the goals of the project but to get someone to come in and do it* (Staff 2)*.’*

*‘My main goal for the trainers is to make sure it’s financially sustainable. Taking away those barriers. I don’t want them to burn out* (Staff 2).*’*

### Recruitment

Tenants were invited to participate in the intervention through an announcement notice which was distributed through the letter boxes of all tenants in the facility. A meeting was held before sessions began in which all tenants who expressed their interest to participate were given an opportunity to learn more about the exercise sessions and what to expect from the intervention. Additionally, facility representatives spread the word by word-of-mouth to tenants, as did the concierge and other staff members affiliated with the project.

*‘It would be good to have a prompt reminder at the start of every week. I forget sometimes* (Week 2).*’*

*‘It’s lovely to be recommended by the concierge* (Week 4)*!’*

### Reach and participation

**Fig. 1:**
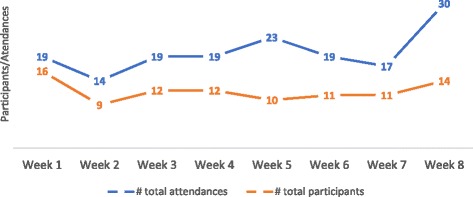
Number of attendances and participants during the intervention period

As outlined in Table 1, reach and participation relates to the target population participation in the intervention and each program session, participant characteristics as well as perceived reasons for non-participation. Exercise physiologist records kept during the 8-week period tracked the total number of participants that signed up during the study period, as well as the attendance per week for these participants, independent of researcher observations. Of the sessions that took place in the 8-week study period, 24 sessions were observed and documented. On average, 2 to 3 participants attended each observed session. There were 2 observed sessions in which no participants attended, and the maximum number of participants to attend was 6. The total number of tenants that participated in the exercise sessions over the 8-week period was 24, and the total number of attendances over this period was 160 with participant attendance ranging from 1 session to 15 sessions with a mean of 6 to 7 (6.666) sessions. These results are shown in Fig. 1*.*

Physical and mental health issues were prevalent in intervention and study participants with all participants having varying levels of physical and mental health conditions, as well as different medical histories. Table 2 presents characteristics of intervention participants including sex, ethnicity, substance use, mental and physical health issues. Of 24 participants 16 (67%) were female, and 8 (33%) were male. 79% (*n* = 19, 12 female) reported having at least one mental health issue, and 75% (*n* = 18, 13 female) indicated physical health issues. 58% (*n* = 14, 11 female) reported substance use.

**Table 2:** Characteristics of intervention participants by sex

	Youngest	Oldest	Mean
Age	24	71	43.7
	Male % (n) 33 (8)	Female % (n) 67 (16)	Total % (n) *n* = 24
Ethnicity			
- Caucasian	75 (6)	75 (12)	75 (18)
- Indigenous Australians	12.5 (1)	18.8 (3)	16.7 (4)
- Other	12.5 (1)	6.2 (1)	8.3 (2)
Mental health issues	87.5 (7)	75 (12)	79.2 (19)
Depression	25 (2)	43.75 (7)	37.5 (9)
Bipolar	12.5 (1)	25 (4)	20.8 (5)
Anxiety	25 (2)	12.5 (2)	16.7 (4)
Schizophrenia	12.5 (1)	6.2 (1)	8.3 (2)
PTSD	0	12.5 (2)	8.3 (2)
Other	25 (2)	12.5 (2)	16.7 (4)
2 or more conditions	12.5 (1)	31.25 (5)	25 (6)
Intellectual disability	12.5 (1)	18.8 (3)	16.7 (4)
Substance use (total)	37.5 (3)	68.75 (11)	58.3 (14)
- Alcohol use (former)	0	12.5 (2)	8.3 (2)
- Alcohol use (current)	12.5 (1)	6.25 (1)	8.3 (2)
- Marijuana	12.5 (1)	6.25 (1)	8.3 (2)
- Smoker	37.5 (3)	56.25 (9)	50 (12)
- Hard drugs^a^	25 (2)	6.25 (1)	12.5 (3)
Physical health issues	62.5 (5)	81.25 (13)	75 (18)
Obesity	12.5 (1)	31.25 (5)	25 (6)
Diabetes	12.5 (1)	18.75 (3)	16.67 (4)
Arthritis	0	25 (4)	16.67 (4)
Chronic pain	12.5 (1)	56.25 (9)	41.67 (10)
Asthma	12.5 (1)	18.75 (3)	16.67 (4)
Hepatitis C	25 (2)	0	8.3 (2)
Other^b^	50 (4)	43.75 (7)	45.8 (11)
2 or more conditions	50 (4)	68.75 (11)	62.5 (15)

^a^e.g. Amphetamines, heroin, solvent sniffing

^b^Liver cirrhosis (1), Graves’ disease (2), peripheral neuropathy (1), hypertension (1), Hiatus hernia (1), epilepsy (1), respiratory disease (1), head injury (1), heart attack (1), significant sensory deficits (1)

Participating in exercise sessions was not always a priority for participants, as they commonly had various personal or family issues that took precedence. Participants expressed concerns about the lack of participation in the intervention from other tenants, and some of their comments implied that the intervention did not reach some of those in need. For example, one of the participants stated that ‘*The people who should be here do not come* (Week 5)*.’* Staff also expressed similar concerns:

*‘Some we saw at the start haven’t come back. For someone who is very resistant, then they’re going to need to change from resistance to contemplation before they’ll even take part* (Staff 3)*.’*

*‘Honestly, sometimes people have bigger things in their life at the moment than going to the gym, even if it would do them a world of good. It’s not a priority for all people* (Staff 4).*’*

*‘For some people it’s a health thing, they don’t think they are healthy enough to participate or they have a physical impairment that causes them pain or difficulty* (Staff 3)*.’*

On the other hand, it was evident from participant comments that those who attended were positive about their participation and had plans to continue.

*‘I’m here to stay. This is the beginning of the new me* (Week 2)*!’*

*‘I wish there were more sessions* (Week 4)*!’*

*‘I feel great. I’m progressing. I’m feeling the benefits. I’m still growing. We’re doing new things every time* (Week 6).*’*

### Dose delivered

Dose delivered component of this evaluation focuses on the extent to which the intended activities, strategies as well as the resources, including equipment and staffing were used. During 8 weeks of intervention, one hour sessions began promptly at 10:00 and 15:00 each day with warm ups, usually involving the bike or mini trampoline. Common activities observed included squats, lunges, push-ups, standing rows, bicep curls, stretches and balance exercises. Depending on participants’ fitness levels, circuits were varied and became more challenging as the weeks progressed for consistently attending participants. Occasionally, exercise physiologists incorporated yoga or pilates routines into sessions. Stretches were commonly completed at the conclusion of fitness sessions. One participant when asked what changes in delivery of the sessions they would like, commented:

*‘Nothing. It’s so great that if I miss a morning session I can just go to the afternoon one* (Week 6)*.’*

#### Trainers

Three qualified exercise physiologists volunteered their time for this intervention. There was always at least one trainer present during a session. Observations revealed that the trainers tailored the exercise routines to individuals’ specific requirements, taking into account their fitness levels and medical histories. They divided their attention amongst clients, setting repetitions of exercises and allocating rest periods, in addition to tailoring to emotional and mental health needs of clients. Trainers constantly supervised participants and provided demonstrations of assigned exercises. According to the trainers, the work delivering the sessions to tenants had a certain degree of unpredictability, which required them to be flexible.

*‘Its hard to know exactly who’s going to turn up given that there’s a variety of different conditions and abilities and barriers to work with. We have to be a bit flexible in each session* (Staff 3)*.’*

Trainers themselves felt they established a good relationship with participants and were impressed by the openness of the participants.

*‘We’ve got good rapport with the clients. They come in and some of them have a great time* (Staff 4)*.’*

*‘It’s been surprising how open participants are to telling their own stories and explain the challenges in their lives* (Staff 3).*’*

#### Equipment

The facility contained a bike, mini trampoline, steps, balls, mats, weights, Total Resistance eXercise suspension equipment, thera-bands, ropes and rollers. As the intervention period progressed, new equipment was added including weights, balls, magic rings and a balance board. Of this equipment, the thera-bands, matts, rollers, balls and mini trampoline were most commonly used. Trainers commented that equipment was very suitable, however, the use of equipment varied with some of it being used regularly and other not used at all due to lower fitness levels of participants.

*‘We use the mats a lot, exercise balls, thera-bands, steps, and the stools. The Total Resistance eXercise suspension equipment we aren’t using, because these people don’t have the strength to use it* (Staff 4).*’*

Interestingly, however, some participants expressed their desire for more intense workouts and to use some of the equipment more frequently:

*‘I’d love to do some more dumbbell work* (Week 4)*.*

*‘I want to do more intense workouts and more cardio* (Week 8)*!’*

### Dose received

Dose received in this study focused on the participant engagement in the activities during fitness sessions, and their reactions to trainers, equipment and other participants. Participation was generally good, and attendees adhered well to trainer instructions. They listened to correction and direction well, and displayed high levels of respect for the trainers. Participants contributed to the positive and encouraging environment. Participants often had different physical conditions that required more attention from trainers; for example, the participant with sensory deficits was more dependent on trainers and needed verbal instruction and constant supervision.

Participants spoke very highly of both exercise sessions and trainers. They commonly expressed appreciation for the individualised attention and care they received. Their comments indicated that trainers played a key role in creating positive and supportive environment. Some of the comments made include:

*‘I enjoy just being able to switch off for an hour and be directed by the trainer* (Week 1).*’*

*‘I love how caring people are here at the sessions compared to most gyms. Here people smile and joke and are friendly, and you can tell they genuinely care about you* (Week 2)*.’*

*‘I’ll take your word for it, I’ve got faith in you* (Week 5)*.’ [addressed to one of the trainers].*

### Satisfaction/feedback

Participants were mostly very enthusiastic and motivated about exercising, and took the sessions seriously. Comments were mainly positive, showing appreciation for the facility and the trainers’ help.

*‘I feel cheerful and positive after today, I feel like I’m rebuilding my life* (Week 1)*.’*

*‘I feel really healthy after sessions. I feel like my body really needed it* (Week 4)*.’*

Participants mentioned a broad range of factors that motivated them to participate including: supportive environment (including proximity of the intervention and the positive/friendly environment within the intervention), motivation by others (by trainer and other participants or building staff), and finally improving wellbeing be it physical or psychological. Example quotes fitting each theme are presented in Table 3 below.

**Table 3:** Factors motivating to participation in the intervention

Supportive environment	*‘Pain. I don’t want it. I want to be fit. Exercise on its own isn’t very exciting. This is so close and convenient- why wouldn’t I come* (Week 5).*’* *‘I can trust that if I’m feeling down if I just get myself to the door (fitness facility) it’ll all be fine. I trust there won’t be any judgement there and there never is* (Week 6).*’* *‘It’s in the building, it’s convenient, it’s a friendly environment, and I like knowing that I’m doing something positive* (Week 8).*’*
Motivation by others	*‘I’ll keep motivating him* (Week 3).*’* [talking about another tenant] *‘It’s lovely to be recommended by the concierge* (Week 4)*!’* *‘This is excellent, working with the master is excellent. He’s so good. I feel like coming again and I’ll make sure I do* (Week 4).*’* *‘[trainer’s name] motivates me to participate when I walk past. He’s very welcoming, and gives me a sense of responsibility for myself* (Week 4).*’*
Improving wellbeing	*‘I’m motivated to participate to increase my strength, work on my arthritis and I want to be able to ride a bike* (Week 1).*’* *‘I’m gonna get a sexy bum* (Week 3)*!’* *‘(…). I want to remain healthy because I have no one to take care of me. I want to live a long life, and I don’t want to be a burden on anyone. You only get one life* (Week 4).*’* *‘I’m motivated to lose weight and become fitter* (Week 4).*’* *‘I always find it brightens me up. It’s slightly harder every time. I can feel myself getting stronger* (Week 5)*.’* *‘I just want to be healthy and do it for myself* (Week 5)*!’* *‘I’ve talked to the doctor, and I gotta lose weight, the doctor recommended it* (Week 5)*.’* *‘I’m motivated by the fear of depression and suicide* (Week 6)*.’*

In addition to improved fitness, participants expressed that the program provided them with additional benefits that extended to other areas of their lives. While some participants enjoyed not being pushed too hard, others stated that pushing themselves through their own limitations was beneficial to them and was transferring into the other areas of their lives.

*‘Exercise makes me less violent and more calm* (Week 3)*.’*

*‘Exercise gives you the resilience to get through tough times* (Week 4)*.’*

*‘I haven’t touched a cigarette in ages* (Week 5)*.’*

*‘I’m pushing through the limitations. It’s transferring into the rest of my life. [personal life event] recently and this has been helping me to handle it better* (Week 6)*.’*

*‘It’s hard road back (from adversity). Illness creates limitations and exercise helps you to push through* (Week 6).’

This is in line with comments made by project staff:

*‘I think that we’ve already had some really great feedback, for example one participant said they were considering committing suicide if it weren’t for us, so that’s a life we’re talking about* (Staff 4)*.’*

*‘We’ve already noticed significant changes in strength, cardiorespiratory, cardio and balance changes with all consumers who come in, so it’s going really well* (Staff 4).*’*

*‘There have been quite a few that have had issues with chronic pain, and every session they seem to come in a bit more optimistic and a bit more comfortable. Certainly, there have been increases (whether its confidence related) in general energy, talking, enthusiasm* (Staff 3)*.’*

### Fidelity

The evaluation element of fidelity refers to the extent to which the intervention implemented was consistent with the underlying theory and philosophy. It was envisaged that the group exercise sessions would be most beneficial to participants due to their potential to broaden social networks and strengthen social connections that this population is lacking, and in turn, improve participant wellbeing. Group sessions were also appropriate given the human resources available.

In delivering the intervention, exercise routines were individually tailored for participants depending on their physical and mental health as well as fitness levels and ability. Trainers frequently asked participants how they felt after different exercises and assessed any pain or discomfort reported. Trainers allocated frequent rest periods and water breaks, ensuring that no participant was pushed too hard. Participant comments also reflected observations as they acknowledged and appreciated their fitness levels being considered.

*‘I enjoy your positivity, and that I don’t get pushed past my limit. You know my limits* (Week 4)*.’*

*‘I have to be careful about exercising because I have (…) disorder. I can’t do all the exercises* (Week 4).*’*

*‘It’s so good here. There’s no judgement and I don’t feel like I’m being pushed too hard* (Week 4)*.’*

*‘I enjoy everything. I can do all the exercises and I hope it helps me lose* weight (Week 5).’

However, the exercise physiologists found that each client required more individualised support than anticipated. This was somewhat contradictory to the initial setup of intervention:

*‘The sessions themselves needed to be a lot more individual focused than was expected, it was anticipated to be more group exercise and inclusive. It is still inclusive, but more individual attention is needed to be given to each participant* (Staff 3).*’*

Due to the identified need for individualised support, unpredictability, and the lack of prior knowledge regarding attendance at the sessions, at times trainers felt understaffed if working on their own.

*‘I think sometimes we’re understaffed, for example if we get 4 people in one session with only [name of the trainer], maybe 1 or 2 are initial consults, how is [the trainer] supposed to do that and attend to (….) the specific and personalised needs of other participants* (Staff 4)*.’*

Existing participant health issues were carefully considered when planning for the intervention to minimise risks and ensure safety and wellbeing of both participants and the trainers. The area that was used for fitness related activities is monitored 24/7 by concierge staff via closed-circuit television. Concierge staff hold mental health first aid certificates. The area has a duress alarm installed that could be used to raise an alarm in the event of a serious incident or injury. During the week at the times the fitness sessions were scheduled there was a nurse who was on duty in the building. Trainers however, pointed out areas for improvement for their own preparedness to work with such a vulnerable population, in particular, managing potential mental health crisis situations.

*‘I think also we have a lack of training in psychology which makes it harder to manage crisis situations. We don’t have enough training in recognizing situations that could escalate, so we don’t have any de-escalation techniques* (Staff 4)*.’*

#### Social interaction

Exercising in a group setting has proved to support social interactions which was positively received by participants. Participants often encouraged each other and interacted well together, communicating freely and asking questions relating to their physical and mental health needs. Participants enjoyed the social interaction; yet occasionally certain participants would cause distractions or demand excessive trainer attention. Participants commented they enjoyed exercising with others and the opportunity for interaction this provided them, as evident in the quotes below:

*‘I love the company when exercising. I can do more than I thought* (Week 2)*!’*

*‘I like participating in groups* (Week 2)*.’*

*‘I enjoy the interaction with other people, the positive environment and the exercise* (Week 8).’

The intervention not only effectively offered opportunities and encouraged social interaction, but also seemed to provide a safe space for participants to open up. According to staff, at times social interaction took priority against exercise:

*‘We’ve also had some sessions where we haven’t done any exercise with the consumer, they’ve simply opened up about their lives, and as an exercise physiologist we see that a lot, because we’re not a psychologist who’s prodding at their mind, we’re an open, fun environment and people are more likely to open up to us* (Staff 4)*.’*

## Discussion

There is limited published research around physical activity interventions for people at risk of homelessness or those who have a history of homelessness. This process evaluation is of a pilot intervention aimed to increase not only physical activity levels, improve physical and mental health, but also encourage social interaction and support among participants and, in turn, help them re-engage with their community. Overall, the process evaluation results indicate that the exercise sessions ran well, and that the exercise room together with the trainers met the requirements for successful implementation. Participants overall, had a very positive experience of this intervention, and indicated it contributed to their physical, mental and social wellbeing. It was evident that the group sessions had benefits due to the social interactions it provided, confirming the program’s holistic benefits and suitability for health promotion of this population. Even though this study did not measure impact, the process evaluation findings indicate such interventions are a worthwhile undertaking for those who aim to improve wellbeing in previously homeless or those who are at risk of homelessness. This study identified several important lessons that need to be considered to ensure the best outcomes and sustainability of projects targeting this population.

One of the key findings was the greater than anticipated need for individualised care for each participant, mainly due to associated various and complex wellbeing issues, which challenged the trainers in delivering the group fitness sessions. This reinforces the need for individualised care when working with such populations, while ensuring participants are getting the benefits from exercising in a group setting, which was one of the key focuses of the intervention. While this was managed in the current intervention; it was relatively short in duration, and the participant levels remained somewhat manageable; this has implications for sustainability of the project. Based on these findings it is clear that working with larger groups would require more trainers to be present at each session, which has implications for the resources required.

Findings of the evaluation highlighted the difficulty of securing funding to implement the intervention. Due to the prominent focus on curative approaches to health as opposed to preventative health approaches, health promotion and public health interventions are often implemented in the context of limited resources. The decreasing funding for preventive health initiatives in recent years, as demonstrated by the most recent Australian federal government Health Budget 2017–2018, illustrates the focus on curative health in Australia. According to the Australian Institute of Health and Welfare Australia’s Health 2016 report, funding for prevention initiatives is now as little as 1.4% of the health budget [35]. However, Australia is not an exception; similar trends can be observed globally in countries such as United Kingdom and United States of America [36, 37]. This certainly has implications for adequate implementation and sustainability of the programs, from which various populations in need could benefit. As a result, smaller projects are often initiated by local champions, recruiting and relying on volunteers’ good will and efforts to improve the wellbeing of others, as was the case in the current project. Consequently, various challenges were faced bringing this project to implementation, starting from setting up the fitness room to sourcing appropriate staff.

With respect to staffing, trainers were central to the success of this intervention. The importance of positive experience with service providers is highlighted in the literature [15, 38]. The trainers established good rapport with participants who unanimously spoke very highly of them. Their ability to create a ‘non-judgemental’, supportive, welcoming and caring environment was highly appreciated and motivated clients to participate and connect well with the trainers. This was a promising finding, given that mistrust is particularly relevant to this vulnerable population, often deterring them from using services [38]. Welcoming experiences in health services among homeless have been linked to feelings of empowerment, being valued as a person and being truly listened to [39]. Convenience of the intervention being on site and free of charge, and overall supportive ‘vibe’ in the supportive housing facility contributed to the development of a supportive environment.

Participants generally enjoyed exercising in a group setting, which also provided opportunities to interact and connect with other participants. These interactions were mainly positive with participants supporting and motivating each other. On the other hand, there were a few concerns regarding some of the participants being distracting. However, participants seemed to focus more on their connection and interaction with trainers, which is not uncommon. Research with people experiencing homelessness indicates that often they do not identify as a collective [40] and this, in turn, may hinder their preparedness to take advantage of opportunities associated with group memberships [15].

The connection between participants and the trainers is important, and might have impact beyond the delivery of the intervention. Previous research indicates the importance of feeling connected to the homeless service and supported by homeless accommodation staff. A study conducted by Johnstone et al. [15] found that those who felt connected to the service and to others described feeling supported and encouraged, and viewed the experience and their situation more positively [15]. The authors also found that experiences at homeless accommodation predicted wellbeing [15] and that these experiences can contribute to the development of multiple group memberships (i.e. social capital). A study by Fitzpatrick et al. [41] found that bridging social capital, or the linking of heterogeneous groups, that links the impoverished to socially dissimilar individuals (trainers could be considered as such in the current study) can help homeless individuals feel better about themselves and their life situation and reduce the odds of suicide ideation [41]. They concluded that social relationships outside the immediate circle of homeless friendships make a difference and can clearly impact one’s health and wellbeing. Bridging social capital appears to give the disadvantaged individual critical access to resources not available within their own social circle. Interestingly, during the interviews of the current study, fear of depression and suicide came up as a motivator to participation. This is consistent with the work of Gregg & Bedard [29], who found that physical activity among homeless shelter patrons was perceived as a method for self-management of mental health issues.

The preceding paragraphs highlight the central importance of trainers to the current intervention, thus warranting further discussion of strategies to recruit and retain suitable staff. The recruitment of staff was a key difficulty of the intervention, identified by participant responses. While investigating trainers’ motivations to volunteer and continuation of their involvement was beyond the scope of this process evaluation, concerns around burnout among the trainers were raised in stakeholder interviews. Burnout and high turnover of staff who work with homeless people is a significant issue discussed in the literature [42]. High staff turnover has been associated with a number of factors, such as: lack of training specific to issues related to homeless, low pay, and overall the difficult nature of work working with vulnerable populations (where traumatic experiences and behavioural health problems are prevalent) [43]. These roles can include engaging with clients while maintaining appropriate boundaries, simultaneously ensuring the safety of clients and themselves, and coping with the emotional strain associated with being exposed to the traumatic life experiences of their clients [43].

Continuity of the trainer’s involvement in the intervention is uncertain if the same voluntary arrangements are to be continued. Issues related to staff working on voluntary basis in similar interventions have been identified in the literature. For example, Gregg and Bedard [29], found that at times, lack of volunteer staff in a fitness facility at a homeless shelter in Canada impacted the continuity of service (i.e. facility was closed when there were no volunteers), which, in turn, was perceived as a barrier to exercise. This indicates the need to either secure funding for human resources, or the combination of both paid and volunteer employees. This would be beneficial as longer term (paid) staff would be well positioned to foster rapport with clients, and minimise stress associated with the unpredictability of participants’ physical and mental limitations. Additionally, it may be beneficial to expand the network of collaborators to include the involvement of educational institutions that prepare trainers. This may increase the pool of trainers willing to participate in the intervention.

Participants expressed concerns about the lack of participation in the intervention from the other supportive housing tenants, and some of their comments implied that the intervention did not reach some of those in need. One of the common and key issues in any public health intervention and research is how to reach those who are most difficult to reach [44–46]. For example, interventions that target youth are commonly implemented in schools and fail to reach those with attendance issues who are often the ones who need the intervention the most [47, 48]. Participants who attended fitness sessions may have been more committed and prepared for a change. This indicates the need for complementary pre-recruitment strategies such as, an on-site fitness expo to promote the value of healthy living. Further, it may well be that those who did not attend sessions are either not interested in fitness and are using other strategies, or have other more pressing priorities that need to be addressed before they can participate in such intervention. Further research investigating reasons for non-attendance could be beneficial to future interventions by increasing participant engagement.

## Conclusion

Group fitness sessions represent a promising intervention to improve wellbeing of this population. The physical activity intervention overall had consistent participation, and received positive feedback from all parties involved. The facility and activities were appropriate for this population, and have been well received by attendees. Being conducted on site, the trainers’ ability to build good rapport with participants together with the supportive environment they created, were central to successful implementation. Findings also revealed a need for more personalised care when delivering fitness sessions, due to the complexity of health issues prevalent in this population. This has implications for already limited resources, including staffing. Strategies to address this are required to ensure the continuity of the program. Given the limited research in this field, this paper reports findings of the implementation of this type of intervention, and is therefore a valuable addition to literature providing insight into how to work with this population. Further research on the impact is required to measure changes/improvements in mental and social health participants may experience as a result of such interventions.
